# A cross-sectional survey on the prevalence of anaemia and malnutrition in primary school children in the Tiko Health District, Cameroon

**DOI:** 10.11604/pamj.2019.32.111.15728

**Published:** 2019-03-11

**Authors:** Egbe Sarah Balle Tabi, Samuel Nambile Cumber, Kenneth Okoth Juma, Elvis Akwo Ngoh, Eric Achidi Akum, Esum Mathias Eyong

**Affiliations:** 1 Center for Research on filariasis and other Tropical Diseases (CRFilMT), Yaoundé, Cameroon; 2 Department of Microbiology and Parasitology, Faculty of Science, University of Buéa, Buéa, Cameroon; 3 Section for Epidemiology and Social Medicine, Department of Public Health, Institute of Medicine, The Sahlgrenska Academy at University of Gothenburg, Gothenburg, Sweden; 4 Faculty of Health Sciences, University of the Free State, Bloemfontein, South Africa; 5 School of Health Systems and Public Health Faculty of Health Sciences, University of Pretoria Private Bag X323, Gezina, Pretoria, 0001, Pretoria, South Africa; 6 African Population Health and Research Center (APHRC), Nairobi, Kenya; 7 Elizabeth Glaser Pediatric AIDS Foundation (EGPAF), Yaoundé, Cameroon; 8 Department of Biochemistry and Molecular Biology, Faculty of Science, University of Buéa, Buéa, Cameroon

**Keywords:** Anaemia, malnutrition, school children, stunting, wasting, underweight, prevalence, predictors

## Abstract

**Introduction:**

Anaemia and malnutrition are common health problems in developing countries with children being the most vulnerable. They have negative impacts on human performance, growth and development, in children, both as cause and consequences of disease. Although annual mass deworming and nutrition supplement strategies have been implemented in the Tiko Health District (THD), no study has been carried out to determine the prevalence of anaemia and malnutrition. The aim of this study was therefore designed to determine the prevalence of anaemia and malnutrition among primary school children aged 5-15 years in the Tiko Health District.

**Methods:**

A cross-sectional study was carried out in 10 randomly selected schools in the THD and a total of 400 school children were enrolled in the study. Body weight and height were measured using an electronic weighing scale and stadiometer respectively. Anthropometric indices: Height-for-Age Z scores (HAZ), Weight-for-Age Z scores (WAZ) and Body Mass Index-for-Age Z scores (BMIAZ) were analyzed and compared with WHO Growth Reference Standards using WHO Anthroplus software. Hemoglobin levels were determined using Urit-12 Haemoglobinometer and anaemia defined as Hemoglobin (Hb) < 11g/dl. Data analysis was done using the SPSS software.

**Results:**

The overall prevalence of malnutrition was 9.25%, prevalence of stunting 7.5% with 0.8% being severely stunted. The prevalence of wasting was 1% and underweight 0.7%. The overall prevalence of anaemia was 5%. Parents occupation and the absence of toilet were statistically associated with anaemia (P = 0.04 and P = 0.003). Age, floor type, absence of toilet and BMI were significantly associated with malnutrition (P = 0.00, P = 0.01, P = 0.02 and P = 0.003).

**Conclusion:**

This study revealed a low prevalence of malnutrition and anaemia which could be attributed to the deworming and nutrition supplement strategies which have been implemented.

## Introduction

Anaemia and malnutrition are one of the most common health problems affecting children. Anaemia affects populations in both rich and poor countries. It is an indicator of both poor nutrition and poor health [1]. Anaemia has been reported as a significant determinant of stunting which is the main type of malnutrition in young children [2]. A 2008 WHO analysis reported that anemia affected 24.8% of the world’s population, including 42% of pregnant women, 30% of non-pregnant women and 47% of preschool children [3]. Most recently, global anemia prevalence was estimated at 29% in pregnant women, 38% in non-pregnant women and 43% in children [4]. Worldwide, anemia is attributed to 3 syndromes: iron deficiency (iron deficiency anemia, hookworm, and schistosomiasis), hemoglobinopathy (sickle cell disorders and thalassemia) and malaria [5]. Although the primary cause of anemia is iron deficiency [1], it is seldom present in isolation [6]. The most reliable indicator of anaemia at the population level is blood hemoglobin concentration, though it does not determine the cause of anaemia. Normal Hb distributions vary with age, sex, and physiological status (such as pregnancy) and Hb levels has been shown to be influenced by age and parasite density [7].

Sub-Saharan has the highest prevalence of hunger. Poor nutrition causes nearly 45% of deaths in children under 5, with one in six children underweight and one in four stunted. India has been reported the country with the highest prevalence of underweight in children [8]. In 2011, Cameroon was classified as a low-income food deficit country (LIFDC) by the UN Food and Agriculture Organization (FAO). A World Bank survey conducted in 2010 further indicated that in Cameroon, 36% of children under the age of five are stunted, 16% are underweight, 7% are wasted and 11% of infants are born with a low birth weight. The North Region has 40.2% and 68.2% prevalence rate of malnutrition and anemia respectively while the South region has a prevalence rate of 33.1% and 73.6% respectively. According to UNICEF, children in Cameroon suffer from malnutrition not because of lack of food but due to poor feeding patterns reason why UNICEF is engaged with helping parents feed their children properly so that the distribution of nutritive supplements would no longer be necessary in the near future.

Malnutrition is a public health concern due to its negative impact on human performance, growth and development, especially in children. It is one of the most important underlying causes of mortality in children in developing countries especially during the first five years of life [9]. It accounts for the death of about 9 million children below 5 years old annually [10] and 50% of deaths in children [8]. A high prevalence of malnutrition has been seen in children in the South West Region of Cameroon [11]. Areas endemic for malaria often have a high prevalence of micronutrient malnutrition.

Malnutrition commonly affects all groups in a community, but infants and young children are the most vulnerable because of their high nutritional requirements for growth and development [11]. Socio-economic, epidemiologic factors and potentially other parasite infections, intertwine to cause anaemia and malnutrition in rural populations [12]. Poor personal hygiene has shown to be associated with anaemia and nutritional deficiency (low BMI) [13]. Health problems due to miserable nutritional status in primary school-age children are among the most common causes of low school enrolment, high absenteeism, early dropout and unsatisfactory classroom performance [14]. Chronic under nutrition in childhood causes slow cognitive development with serious health impairments later in life that reduce the quality of life of individuals. Nutritional status being an important index of this quality, anthropometric examination is a mandatory tool to assess health and nutritional condition in children [8]. Under nutrition in children is classified using three categories: underweight, stunting and wasting [14, 15].

Stunting is defined as a low Height-for-Age for children and it measures the past (chronic) child under nutrition. Stunting is frequently associated with repeated exposure to adverse economic conditions, poor sanitation and interactive effects of poor energy, nutrient intake and infection. Stunting reflects failure to receive adequate nutrition over a long period of time and is affected by recurrent and chronic illness. A child is considered stunted if the child is too short for his/her age [6]. Children with z-scores < - 2.00 are said to be stunted and those < - 3.00 are severely stunted.

Wasting is defined as low Weight-for-Height or low BMI for age for children, measuring current or acute under nutrition and is closely tied to mortality risk. It is generally associated with recent illness, weight loss or a failure to gain weight [9]. A child is considered wasted if the child is too thin or weighs too little for his/her height. The weight-for height indicator also can be used to assess the extent to which children are overweight or obese, which is an increasing problem among children worldwide [6]. Children with z-scores < - 2.00 are said to be wasted and those < -3.00 are severely wasted.

Underweight is defined as low weight-for-age and it reflects past (chronic) and present (acute) under nutrition. Low weight-for-age indicates a history of poor health or nutritional deficiencies, including recurrent illness and/or starvation. Weight-for-Age indicates an assessment of whether a child weighs too little for his/her age. A child can be underweight for his/her age because the child is stunted, wasted or both [6]. Children with z-scores < - 2.00 are said to be underweight and those < -3.00 are considered as severely underweight. As a result of its inability to differentiate between relative height and body mass, weight-for-age is not recommended for the assessment of growth beyond childhood (> 10 years of age) [16].

The nutritional status of children does not only reflect the socio-economic status of the family and social wellbeing of the community, but also the efficiency of the health care system and the influence of the surrounding environment. Nutritional status of school age children is very important since the foundation of lifetime health and intellectual vitality is laid during that period [8]. Regular updates on the nutritional status of school children is very important though data is lacking reason why this study was carried out to determine the prevalence of anaemia and malnutrition in primary school children in the Tiko Health District.

## Methods

### Study area and population

**Figure 1:**
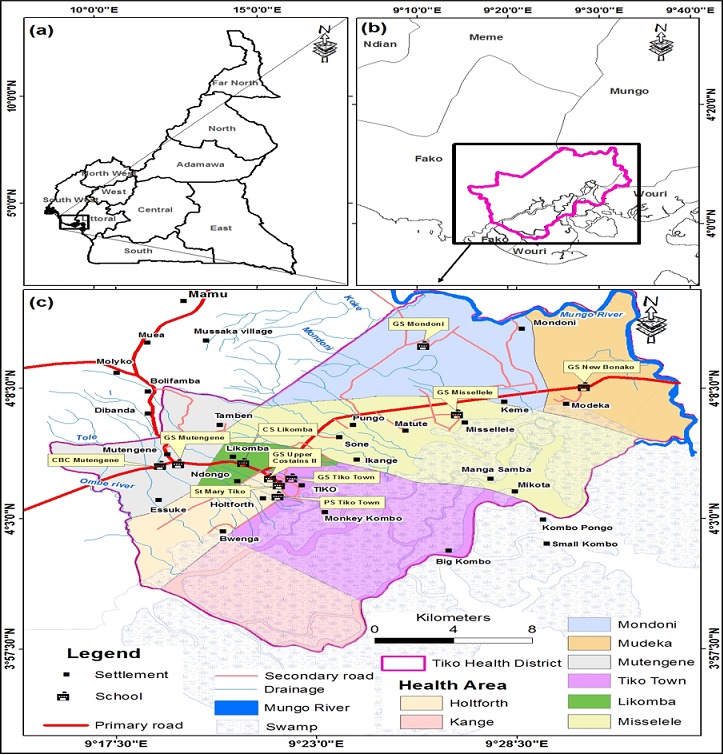
Geographic location of primary schools selected for the study in the Tiko Health District

This study was carried out in the Tiko Health District, located in Fako Division in the South West Region of Cameroon. The district has a total surface area of 484 km^2^ and located between longitude 8.6°10’E and latitude 4°5.2’N. Tiko has a coastal equatorial climate with daily temperatures ranging from 28°C to 33°C. Soil types include the sandy alluvial and volcanic with high agricultural potentials. The main water courses in the Tiko municipality include River Mungo, the Ombe River, Ndongo and Benyo streams which empty into the Atlantic Ocean. The main activity of the population is trading, fishing, livestock and industrial agriculture. The THD is bounded in the North by Buea, South by Bonaberi, West by Limbe and East by Dibombari. The THD is headed by the District Medical Officer (DMO) and it is made up of 8 Health Areas (HA) namely: Holforth, Kange, Likomba, Mutengene, Mondoni, Mudeka, Missellele and Tiko Town (Figure 1). The THD is endemic for soil transmitted helminths which could be attributed to poor environmental hygiene and sanitation and lack of adequate water supplies. The THD has 84 primary schools including public (31), private lucrative (29) and private confessional schools (24) (IBE Tiko statistics - 2016). It has a total population of 147,423 and 12.02% (17,722) of this population are primary school children. This study comprised of primary school children aged 5-15years in the THD who were present at the time of the study.

### Study design, inclusion criteria

A cross sectional study was carried out from April to May 2016, to determine the current prevalence of anaemia and malnutrition among primary school children in Tiko Health District. Children were included in the study if their parents/legal guardians gave their consent by signing the informed consent forms and if the children gave their assent and were voluntarily willing to participate in the study. Children who were sick or suffering from severe medical conditions were excluded from the study.

### Sampling method and sample size determination

A stratified cluster sampling technique was used to recruit the study participants. The 84 primary schools found in the THD are unevenly distributed among the 8 HAs. In HAs with less than 7 schools, just one primary school was randomly selected while in HAs with more than 7 schools, two primary schools were randomly selected. Random selection was done by writing the name of each school with respect to the HA on a separate piece of paper, which was then placed in a box and thoroughly mixed before selection. A simple random sampling technique was applied by blindly picking one or two papers where needed and the name of the selected school(s) written in a field note book. A total of 10 schools were selected and informed parental consents were given to all children present from grade 1 to 6, for information and written approval for their children to participate in the study. A sample size of 400 was used for the study.

### Data collection

Prior to the start of the study, visits were made to all the randomly selected schools. Letters of administrative authorizations were presented to the various head teachers and the purpose/benefits of the study explained to both the teachers and children. Children present on the day before sample collection were given consent forms to take to their parents/legal guardians to read and consent by signing. Children were identified by individual codes and their names written separately in a notebook for the purpose of the return of their results. This study included the collection of both qualitative and quantitative data.

### Anthropometric measurements

The height and weight of each child was measured to determine their anthropometric indices. Weight was measured without shoes and with minimum clothing, using an electronic weighing scale to the nearest 0.1 kilogram (kg). Height was measured to the nearest 0.1centimeters (cm) in bare feet with participants standing upright against a mounted stadiometer. Both measurements were recorded in the individual’s questionnaire. Weight-for-Age Z-score (WAZ), Height-for-Age Z-score (HAZ) and Body Mass Index-for-age Z-score (BMIAZ) were calculated to assess underweight, stunting and wasting status respectively, as indicators of under nutrition. Age, sex, height and weight were used to evaluate nutritional status. WAZ, HAZ and BMIAZ were compared with WHO Growth Reference Standards using anthroplus open access software (Anthro-Plus v1.0.4, WHO, Geneva, Switzerland). Children were classified as underweight, stunted or wasted respectively, when their Z-scores fell two or more standard deviations below the mean.

### Determination of hemoglobin levels

Blood collection was done by finger prick using disposable droplet lancets and 15µl of blood was collected to measure hemoglobin (Hb) using a URIT-12 hemoglobin meter (URIT, made in China). The tip of the middle finger of the left hand was cleaned with a cotton swap soaked in 70% alcohol and then pricked with the lancet. The first drop of blood was cleaned and the subsequent drop placed on the sample spot of the H12 hemoglobin test strip inserted into the hemoglobin meter. After 20 seconds, the Hb value displayed in g/dl was recorded in the data section of the individual’s questionnaire. Anaemia was defined as Hb level < 11g/dl (Pasricha *et al.*, 2014) and Hb concentrations < 7g/dl, 7.0 - 9.9g/dl and 10.0 - 10.9g/dl classified as severe anemia, moderate anaemia and mild anaemia respectively [17]. Results of children who were anaemic were immediately issued through the class teacher to their parents for immediate further action and clinical management.

### Collection of geo-spatial data

Geographic coordinates (latitudes and longitudes) of each surveyed school in the respective health areas were taken using a Global Positioning System (GPS) application downloaded on a smartphone. These coordinates were recorded in a field note book.

### Statistical analysis

After data editing, the primary data collected was fed into Microsoft Excel 2013 spreadsheet and then exported to SPSS (v.20) for analysis. Data analysis was done by running descriptive statistics and cross tabulations. Graphs were drawn using Microsoft Excel 2013. Test for normality (Kolmogorov) was used to establish if the variables were normally distributed. Chi square test was used to investigate the association between prevalence of anaemia, malnutrition and age, gender. Relationship between independent and dependent variables was assessed by chi-square test. Univariate and multivariate logistic regression models were used to assess the association between anaemia, malnutrition and predictor variables. Anthropometric indices, HAZ, WAZ and BMIAZ scores were calculated using WHO Anthro Plus software 2009.

### Ethical considerations

Ethical clearance was gotten from the Institutional Review Board of the Faculty of Health Science, University of Buéa, Cameroon. Administrative authorizations were gotten from the Regional Delegation of Public Health and Regional Delegation of Basic Education in the South West Region of Cameroon. Additional administrative approvals were gotten from the District Medical Officer of the Tiko Health District, Inspectorate of Basic Education Tiko and Head teachers of the selected schools. A brief talk was given to the school children on the objectives, protocol and benefits of the study. Participation in the study was voluntary, children’s assent was sought and only the children whose parents consented by signing the informed consent form were recruited as study participants. Participants had equal chances of participating in the study and had the right to withdraw at any time during the study without being questioned. Confidentiality was ensured by giving serial numbers to each participant. Participant’s names were only used for the purpose of issuing of individual results. Capillary blood samples were collected by trained nurses and medical laboratory scientists with experience in phlebotomy and pediatrics. At the end of the study, all the selected schools were revisited and the results of each participant was given. Head teachers of schools with anaemic children were properly informed and had the responsibility of informing the parents of these children, to consult at the health centre to ensure proper management of their clinical condition.

## Results

### Socio-demographic characteristics of the study participants

Of the 400 study participants, 183 (45.7%) were males. Majority of the study participants, 50.7% were aged between 5-9 years and 49.3% > 9 years. The median age of the study population was 9 (IQR = 3), with an age range between 5-15 years spread across grade 1 to grade 6. Grades 1 had the lowest proportions (2.3%) of study participants while Grade 5 had the highest proportion of participants (40.5%). Majority of the parents of the children (36.5%) were employed by the Cameroon Development Corporation (CDC) while some were involved in business (19.5%). The least proportions were found among the parents involved in fishing (2%). A greater proportion of the participants (66.3%) lived in block houses and a least proportion of children lived in asbestos (7%) (Table 1).

**Table 1:** Socio-demographic characteristics of the study population

Variables	Frequency (n)	Percentage (%)
**Gender**		
Male	183	45.7
Female	217	54.3
**Age (years)**		
5-9	203	50.7
>9	197	49.3
**Grade**		
1	9	2.3
2	17	4.3
3	88	22
4	99	24.8
5	162	40.5
6	25	6.3
**Parents occupation**		
Farmer	57	14.3
Fishing	8	2.0
Business	78	19.5
Civil Servant	25	6.3
CDC	146	36.5
Unemployment	14	3.5
Others	72	18.0
**House type**		
Cement	265	66.3
Planck	107	26.8
Asbestos	28	7.0
Cemented	326	81.5
Uncemented	74	18.5

### Prevalence of anaemia

**Figure 2:**
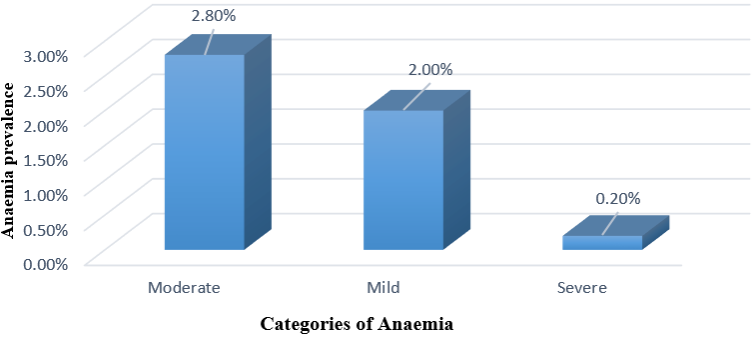
Categories of anaemia in the study population

The median Hb level in the study was 14.4g/dl, while the Interquartile Range (IQR) was 2.4 (95% CI = 14.3, 14.6). The minimum and maximum Hb levels recorded were 6.7g/dl and 19.9g/dl respectively. The prevalence of anaemia (Hb < 11g/dl) in school children was found to be 5% (95% CI = 2.86 - 7.14). Out of the 20 children who were anaemic, the prevalence’s of severe anaemia (Hb < 7 g/dl) was found to be 0.2% (95% CI = 0 - 0.74), mild anaemia (Hb 7.0 - 9.9 g/dl) was 2% (95% CI = 0.63 - 3.37) and moderate anaemia (Hb 10.0 - 10.9g/dl) was 2.80% (95% CI = 1.15 - 4.35) (Figure 2). Hb levels were analyzed by gender, age group and infection with Soil-transmitted helminths (STH). It was observed that female school children had a higher prevalence of anaemia (5.5%) than male school children (4.4%). But this observed difference was statistically not significant (χ^2^ = 2.58, P = 0.46). Children aged between 5-9 years of age had a higher prevalence of anaemia (6.6%) than those aged > 9 years (3.5%). However, the observed difference between age groups was found not to be statistically significant (χ^2^ = 5.36, P = 0.14).

### Nutritional characteristics of the study participants

All of the 400 study participants had complete anthropometric measurements. The mean ± SD of HAZ, WAZ and BMIAZ scores were - 0.50 ± 1.04, -0.11 ± 0.78 and 0.03 ± 0.83 respectively. The overall prevalence of malnutrition in the study population was 9.25% (95% CI = 6.8 to 12.3%) with stunting being the most common form (8.3%, CI = 5.0 to 12.1%), followed by wasting (1%, CI = 0.3 to 2.0) and underweight (0.7%, CI = 0.0 to 1.8%) (Table 2).

**Table 2:** Nutritional characteristics of the study participants

Variables	Mean ± SD	Median	IQR	Range	95% CI
Weight in kg	29.17 ± 6.87	27.6	7.4	[14.1-7.4]	27.15 to 18.15
Height in cm	131.57 ± 10.02	130	13	[100-165]	129.0 to 132.0
Hb level in g/dl	14.28 ± 1.91	14.4	2.4	[6.7-19.9]	14.3 to 14.6
BMI	16.65 ± 1.97	16.4	2.1	[12.9-29.2]	16.25 to16.60
WAZ	-0.11 ± 0.78	-0.11	0.97	[-2.54-2.63]	-0.20 to -0.02
HAZ	-0.50 ± 1.04	-0.49	1.37	[-3.51-2.39]	-0.60 to -0.40
BMIAZ	0.03 ± 0.83	0.09	1.03	[-2.99-2.91]	-0.05 to 0.11
**Variables**	**%**	**95% CI**			
Prevalence of malnutrition	9.25	--	--	--	6.8 to 12.3
Prevalence of underweight	0.7	--	--	--	0.0 to 1.8
Prevalence of stunting	8.3	--	--	--	to 12.1
Prevalence of wasting	1	--	--	--	0.3 to 2.0

### Prevalence of malnutrition

Of the 400 school children surveyed, 37 were malnourished giving an overall prevalence of 9.25% (95% CI = 6.8 to 12.3). The overall malnutrition status was analyzed with respect to gender, age, STH infection status and anaemia status. It was observed that most of the malnourished children were amongst the age group > 9 years (15.7%) with just 6 cases of malnourished children in the age group 5 to 9 years. A statistically significant difference was observed in the prevalence of malnutrition between the age groups (χ^2^ = 19.45, P = 0.00). Analyzing by gender, it was found that the prevalence of malnutrition was almost similar between males (9.8%) and females (8.8%). There was no statistically significant difference between gender and malnutrition (χ^2^ = 0.14, P = 0.71). Analyzing by anaemia status, most of the malnourished children were non anaemic (9.3%) and just one anaemic child was reported to be malnourished. There was no statistically significant difference observed between anaemia status and malnutrition (χ^2^ = 0.45, P = 0.50).

### Underweight in primary school children

Weight-for-Age Z-scores were only computed for school children 5 to 10 years of age (n = 284). The mean weight-for-age Z-score reported in the study was -0.11 ± 0.78 (95% CI= -0.20 to -0.02). Minimum and maximum Z-scores were -2.54 and 2.63 respectively. The overall prevalence of underweight (WAZ = - 2) was 0.7% (95% CI = 0.0 to 1.8). No case of severe underweight was reported. Weight-for-Age Z-scores were analyzed by age group, gender, anaemia status and infection with STH. It was observed that the only 2 cases of underweight were amongst the 5-9 years age group and no case of underweight was reported in the > 9 years age group. However, there was no statistically significant difference observed between the age groups (χ^2^ = 0.80, P = 0.37). Analyzing by gender, there was an equal prevalence of underweight in both male (50%) and female (50%) school children, but there was no statistically significant difference observed between gender and underweight (χ^2^ = 0.03, P = 0.85). Analyzing by anaemia status, the 2 cases found to be underweight were non anaemic while the 16 who were anaemic had normal Weight-for-Age Z-scores. However, there was no statistically significant difference observed between anaemia status and underweight (χ^2^ = 0.12, P = 0.73) (Table 3).

**Table 3:** Prevalence of underweight by age, sex and anaemia status of the study population

Variables		Underweight		Total	χ	P-value
		Normal n [%]	Underweight n [%]	N		
**Age [Years]**	5-9	201 [99]	2 [1]	203	0.80	0.37
	>9	81 [100]	0	81		
Total		282	2	284		
**Sex**	Male	123 [99.2]	1 [0.8]	124	0.03	0.85
	Female	159 [99.4]	1 [0.6]	160		
Total		282	2	284		
**Status**	Anaemic	16 [100]	0	16	0.12	0.73
	Non anaemic	266 [99.3]	2 [0.7]	268		
Total		282	2	284		

### Stunting in primary school children

Height-for-Age Z-scores were computed for all the school children 5 to 15 years of age (n = 400). The mean Height-for-Age Z-score reported in the study was - 0.50 ± 1.04 (95% CI = -0.60 to -0.40). Minimum and maximum Z-scores were - 3.51 and 2.39 respectively. Stunting (HAZ = - 2) was the highest form of malnutrition reported in the study, with an overall prevalence of 8.3%. Stunting was observed in 30 children (7.5%) (95% CI = 5.0 to 10.3) and severe stunting (HAZ = - 3) in 3 children (0.8%) (95% CI = 0.0 to 1.8). Height-for-age Z-scores were analyzed by age group, gender, anaemia status and STH infection status. A higher rate of stunting was observed amongst the age group > 9 years (14.2%) with the 3 cases of severe stunting recorded within this age group. A lower rate of stunting was observed amongst the age group 5-9 years (2.5%). There was a statistically significant difference in the prevalence of stunting between the age groups (χ^2^ = 18.53, P = 0.00). Analyzing by gender, it was found that the prevalence of stunting in male school children was 9.8% and that in female 6.9%. There was no statistically significant difference between gender and stunting (χ^2^ = 3.86, P = 0.14). Furthermore, it was observed that male children had a higher prevalence of severe stunting (1.6%). With regards to anaemia status, there was a higher prevalence of stunting in the non anaemic group (8.4%) as compared to the anaemic group (5%). However, there was no statistically significant difference observed between anaemia status and stunting (χ = 0.36, P = 0.84) (Table 4).

**Table 4:** Prevalence of stunting by age, sex and anaemia status

Variables		Stunting			Total	P-value
		Normal n [%]	Stunted n [%]	Severe Stunting n [%]	N	
**Age [Years]**	5-9	198 [97.5]	5 [2.5]	0	203	0.00**
	>9	169 [85.8]	25 [12.7]	3 [1.5]	197	
Total		367	30	3	400	
**Sex**	Male	165 [90.2]	15 [8.2]	3 [1.6]	183	0.14
	Female	202 [93.1]	15 [6.9]	0	217	
Total		367	30	3	400	
**Status**	Anaemic	19 [95]	1 [5]	0	20	0.84
	Non anaemic	348 [91.6]	29 [7.6]	3 [0.8]	380	
Total		367	30	3	400	

** Statistically significant, p < 0.05

### Wasting in primary school children

BMI-for-age Z-scores were computed for all the school children 5 to 15 years of age (n = 400). The mean BMI-for-age Z-score reported in the study was 0.03 ± 0.83 (95% CI = - 0.05 to 0.11). Minimum and maximum Z-scores were - 2.99 and 2.91 respectively. Wasting (BMIAZ = - 2) was the second highest form of malnutrition reported in the study, with an overall prevalence of 1%. Wasting was observed in 4 children (1%) (95% CI = 0.3 to 2.0). Severe wasting was absent in the study population. BMI-for-age Z-scores were analyzed by age group, gender, anaemia status and STH infection status. A higher rate of wasting was observed amongst the age group > 9 years (1.5%) as compared to the age group 5-9 years who had a lower rate of wasting (0.5%). There was no statistically significant difference in the prevalence of wasting between the age groups (χ^2^ = 1.07, P = 0.30). It was observed from the study that the prevalence of wasting was higher in females (1.8%) with no male being wasted (0%). However, there was no statistically significant difference between gender and wasting (χ^2^ = 3.41, P = 0.06). Analyzing by anaemia status, there was a higher prevalence of wasting in the non anaemic group (1%) as compared to the anaemic group (0%) and all the 4 cases reported as wasted were non anaemic. There was no statistically significant difference observed between anaemia status and wasting (χ^2^ = 0.21, P = 0.64) (Table 5).

**Table 5:** Prevalence of wasting by age, sex and anaemia status

Variables		Wasting		Total	χ	P-value
		Normal n [%]	Wasted n [%]	N		
**Age [Years]**	5-9	202 [99.5]	1 [0.5]	203		
	>9	194 [98.5]	3 [1.5]	197	1.07	0.30
Total		396	4	400		
**Sex**	Male	183 [100]	0	183		
	Female	213 [98.2]	4 [1.8]	217	3.41	0.06
Total		396	4	400		
**Status**	Anaemic	20 [100]	0	20		
	Non anaemic	376 [98.9]	4 [1]	380	0.21	0.64
Total		396	4	400		

### Risk factors associated with the development of anaemia

Examining the association of anaemia with the possible risk factors, it revealed that parent’s occupation and the absence of toilet were significantly associated with anaemia (P = 0.04, P = 0.003). Multivariate analysis using logistic regression confirmed that, the absence of toilet (AOR = 6.38; 95% CI = 1.88, 21.6), and parents occupation (AOR = 5.14; 95% CI = 0.65, 40.07) were the main predictors of anaemia among the study subjects. Participants without toilets were 6.38 times more likely to develop anaemia (Table 6).

**Table 6:** Risk factors associated with anaemia in the study population

Independent Variables		Multivariate Logistic Regression Dependent Variable Anaemia		
		COR [95%CI]	AOR [95%CI]	P value
**Age [Years]**	5-9	1	1	0.16
	>9	0.76[0.23-2.50]	0.49[0.18-1.33]	
**Gender**	Male	1	1	0.69
	Female	1.11[0.40-3.08]	1.20[0.46-3.12]	
**Parents occupation**	Farmer	1	1	0.04**
	Fishing	6.11[0.67-55.03]	5.14[0.65-40.07]	
	Business	0.51[0.08-2.93]	0.74[0.14-3.87]	
	CDC	0.29[0.05-1.60]	0.32[0.06-1.64]	
	Unemployed	2.03[0.23-17.5]	2.27[0.31-16.44]	
	Others	1.48[0.32-6.82]	1.70[0.39-7.24	
**Hand wash before eating**	Always	1	1	0.14
	Sometimes	3.87[1.16-12.92]	2.28[0.75-6.85]	
**Toilet present**	Yes	1	1	0.003**
	No	6.97[1.77-27.42]	6.38[1.88-21.6]	
**Finger biting**	Yes	1	1	0.12
	No	2.14[0.77-5.97]	2.10[0.80-5.53]	
**Source of water**	Tap	1	1	0.20
	Well	1.17[0.12-11.24]	0.36[0.07-1.73]	

** Statistically significant [p <0.05], COR: crude odd ratios, AOR: adjusted odd ratios

### Risk factors associated with the development of malnutrition

Multivariate logistic regression was used to assess predictors of malnutrition among the study subjects. Age (AOR 9.72, 95% CI, 3.67-25.76), floor type (AOR 2.72, 95% CI, 1.24-5.98), toilet absent (AOR 3.19, 95% CI, 1.17-8.69) and BMI (AOR 0.78, 95% CI, 0.62-0.97) were positively associated with malnutrition among the study participants (Table 7).

**Table 7:** Risk factors associated with malnutrition in the study population

Independent Variables		Multivariate Logistic RegressionDependent VariableMalnutrition		
		COR [95% CI]	AOR [95% CI]	P value
Age	5-9	1	1	0.00**
	>9	10.06 [3.67-27.5]	9.72 [3.67-25.76]	
Gender	Male	1	1	0.95
	Female	1.01 [0.48- 2.13]	0.98 [0.47-2.02]	
Floortype	Cemented	1	1	0.012**
	Uncemented	2.72 [1.14-6.52]	2.72 [1.24-5.98]	
Toilet present	Yes	1	1	0.023**
	No	2.86 [0.96-8.48]	3.19 [1.17-8.69]	
BMI		0.77 [0.62-0.97]	0.78 [0.62-0.97]	0.027**

** Statistically significant, p <0.05, COR: crude odd ratios, AOR: adjusted odd ratios

## Discussion

Our study indicated an anaemia prevalence of 5% which is similar to the prevalence obtained in Ekona, Cameroon (6%) by Kimbi and others [18]. However, a lower prevalence rate (1%) was reported in an urban area by Kimbi *et al.* (2012), in school age children in Honduras (2.2%) [16] and in school children in Tanzania (3.1%) [6]. Several studies have reported high prevalence rates of anaemia such as: 71.5% at a baseline study in children 6 months to 10 years living in Mutengene, South West Region of Cameroon [19], 44.8% in children aged 10 years and below in Muyuka, Cameroon [20], 19.8% by Sumbele *et al.* in primary school children in the Mount Cameroon area [2], 44.2% [21], 11% [13] and 19.5% [17]. The relatively low prevalence of anaemia (5%) gotten in this study could be as a result of reduction in malaria transmission due to the use of bed nets and effective treatment with Artemisinin-based Combination Therapy (ACTs), as malaria is one of the major causes of anaemia. Secondly, effectiveness of mass drug administration to fight helminths may also have contributed to the low prevalence of anaemia. It is known that females tend to be more anaemic than males as a result of physiological differences and this was observed in this study. However, there was no significant difference in the prevalence of anaemia between males and females and this is consistent with the findings of Munisi *et al.* (2014), Tchinda *et al.* (2012) [22], who found out that there was no difference in the prevalence of anaemia between gender and age groups. In contrast to a previous study in Northern Rwanda which demonstrated a high prevalence of anaemia among the male compared to the female school children [23]. This study also indicated that children in the older age groups were less likely than their counterparts in the lower age groups to be anaemic. This finding might be explained by the fact that children have high iron demand at early childhood [23].

The overall prevalence of malnutrition was 9.25% which is almost similar to the findings of Sanchez and others who found a prevalence of 10.3% in school age children in a rural community in Honduras [16]. The findings in this study were in contrast with the results of a study carried out among Cameroonian populations [11] which reported a high rate of malnutrition (58.1%) and another study carried out by Sumbele and colleagues, who found a prevalence of 22.8% in children in Muea [15]. Stunting was the most common type of malnutrition found in this study which is consistent with the findings of Sumbele *et al.* [15] and Nkuo-Akenji *et al.* who found stunting to be more common than underweight and wasting, likely reflecting the low socio-economic status of the inhabitants [11]. Higher prevalence of stunting and thinness has also been reported in Ethiopia [13] and Tanzania [6]. The present study observed that there was no significant difference in the prevalence of thinness between male and female, results which are consistent with previous report by Munisi and colleagues [6]. The low prevalence of underweight (0.7%) in this study is consistent with the findings reported from a previous study (1.3%) [16]. Since malnutrition reflects the low socio-economic status of individuals or households, poverty is therefore linked to their dietary intake. This study showed that children below the ages of 10 years old were significantly less stunted as compared to those ≥ 10 years. This is consistent with a similar study carried out in rural Malaysia [24]. Stunted children continue to deviate from normal growth with increasing age and so the risk of becoming stunted continues as children get older. In this study, male children were more stunted than the females and this has been reported in other studies [6, 22]. Male children may be at increased risk of stunting because they tend to be more active, running around in the neighborhood and not always available as compared to female children who are always available to eat whatever food that their mothers provide for them.

The predictors of anaemia were parents’ occupation and the absence of toilet while for malnutrition it was age, floor type, absence of toilet and BMI. This is consistent with the findings of a study carried out by Mahmud *et al.* (2013) who found that children without latrine were at risk of becoming anaemic and malnourished. Not much difference was observed between the crude and adjusted odds ratios, indicating that the observed associations were not affected by age and gender of the child.

## Conclusion

Based on the findings of this study, we therefore conclude as follows; the prevalence of anaemia in the Tiko Health District was 5%, prevalence of malnutrition was 9.25%, the risk factors associated with the development of anaemia were parents’ occupation and the absence of a toilet. Risk factors to malnutrition were age, floor type, BMI and the absence of a toilet. **The strengths of the study:** this study had a representation of the Health Areas in the Tiko Health District and made use of a large sample size; all of the 400 study participants had complete anthropometric measurements. **The weaknesses of the study:** other factors associated with the occurrence of anaemia such as malaria, iron deficiency, micronutrient deficiencies and haemoglobinopathy were not measured and taken into consideration; with regards to sampling of schools, this study did not take into consideration the schools located in the urban and rural areas of the district as done by other studies; no baseline data on the prevalence of anaemia and malnutrition in the THD. **Unanswered questions and future research:** studies should be carried out to determine the factors associated with the observed prevalence of anaemia and malnutrition among primary school children in THD so that appropriate control measures could be planned and implemented: systematic screening for malnutrition should be carried for all school children; despite deworming and nutrition supplement strategies, the results of this suggest that socio-economic and epidemiologic factors, and potentially other parasite infections, intertwine to cause anemia and malnutrition in this population. Hence further studies should be carried out to address this factors; further studies should be carried out with a larger sample size and more exhaustive evaluation of other factors like education, micronutrients, parasitic infection.

### What is known about this topic

- Several studies have reported on anaemia and malnutrition in school children such as: a community-based longitudinal study carried out in a cohort of 357 children aged 6 months to 10 years living in Mutengene, south-western region of Cameroon to determine the prevalence of anaemia;
- Burden of malnutrition among children less than 14 years of age in a rural village of Cameroon;
- Burden of under nutrition in schoolchildren residing in Mfou health district in Cameroon.

### What this study adds

- Despite limitations, the study provides data on the current prevalence of anaemia and malnutrition which forms a basis for evaluation of the control program and highlights the need for further research and interventions to improve the studied indicators;
- This study establishes the fact that there is need for evaluations of micronutrient supplementation strategies and deworming exercises carried out in this population;
- This study also brings out the importance of hygiene and sanitation especially with regards to the availability and usage of toilets.

## Competing interests

The authors declare no competing interests.
